# Plant-Derived Protein Hydrolysates and Bioactive Peptides for Restoring Gut Health in Undernutrition: A Scoping Review of Intestinal Barrier Protection, Inflammatory Signaling, and Oxidative Stress Regulation

**DOI:** 10.3390/nu18183094

**Published:** 2026-09-21

**Authors:** Asad Abbas, Muhammad Khurram Afzal, Fahid Nazir, Ralf Weiskirchen, Muhammad Bilal, Ghalia Arshad, Farah Aftab, Elsa Malik, Shazia Akram, Nudrat Khursheed, Bipindra Pandey

**Affiliations:** 1 Department of Human Nutrition, Faculty of Food Science and Nutrition, Bahauddin Zakariya University, Multan 60800, Pakistan; asadabbaskhichi@gmail.com (A.A.); ghaliaarshad0@gmail.com (G.A.); farahaftab748@gmail.com (F.A.); shaziaakram2001@gmail.com (S.A.); nudratkhursheed786@gmail.com (N.K.); 2 Multan College of Nutritional Sciences, Multan Medical and Dental College, Multan 60000, Pakistan; elsamalik72@gmail.com; 3 Department of Human Nutrition and Dietetics, University of Management and Technology, Sialkot Campus, Sialkot 51310, Pakistan; fahid.nazir@skt.umt.edu.pk; 4 Institute of Molecular Pathobiochemistry, Experimental Gene Therapy and Clinical Chemistry (IFMPEGKC), RWTH University Hospital Aachen, D-52074 Aachen, Germany; 5 Department of Food Science and Technology, Faculty of Food Science and Nutrition, Bahauddin Zakariya University, Multan 60800, Pakistan; sheikhbilal4666@gmail.com; 6 Department of Pharmacy, Madan Bhandari Academy of Health Sciences, Hetauda 44100, Bagamati Province, Nepal; bipindra.pandey@mbahs.edu.np

**Keywords:** plant-derived peptides, protein hydrolysates, gut microbiota, intestinal barrier, childhood undernutrition, oxidative stress

## Abstract

**Background:** Childhood undernutrition is associated with intestinal barrier dysfunction, gut dysbiosis, and increased susceptibility to gastrointestinal complications. This review examines whether plant-derived protein hydrolysates and bioactive peptides can influence gut barrier integrity, inflammatory signaling, oxidative stress, nutrient absorption, and microbiota composition. **Methods:** A scoping review of evidence published from 2010 and 2026 was conducted following a structured, multi-database search and screening process. Studies were assigned to defined evidence categories according to whether the intervention was an identified peptide sequence, a peptide fraction, a whole hydrolysate, an intact protein, or a complex food matrix. **Results:** Plant-derived protein hydrolysates, peptide fractions, and identified peptides were reported to increase occludin, claudin-1, and ZO-1 expression and reduce epithelial permeability in colitis models and in human-derived intestinal monolayers. Several interventions inhibited NF-κB and MAPK signaling, reducing TNF-α, IL-6, and IL-1β. Selected interventions lowered intestinal malondialdehyde and raised superoxide dismutase, glutathione peroxidase, and total antioxidant capacity; three studies demonstrated Keap1–Nrf2–ARE pathway activation directly. Microbiota effects were compositional, with increased short-chain fatty acid production, higher abundances of *Lactobacillus* and *Bifidobacterium*, and reduced pathobionts. Evidence for nutrient absorption was weaker: no included study measured PEPT1 expression, transport activity, or peptide flux following hydrolysate administration, and most sequences reviewed exceeded the di- and tripeptide substrate limit of that transporter. **Conclusions:** No included study administered a plant-derived peptide in a model of undernutrition, and none assessed clinically diagnosed SIBO. Mechanistic evidence suggests plausible benefits for gut barrier integrity, redox balance, inflammation, and microbiota composition, but controlled trials in undernourished children are required before clinical claims can be made.

## 1. Introduction

Childhood undernutrition remains a major global health concern, with an estimated 45 million children under 5 years of age affected by wasting and 149 million by stunting [1]. Its consequences extend beyond inadequate nutrient intake, increasing mortality risk and contributing to chronic growth retardation, impaired immune function, and poor brain development [2]. The burden is highest in low- and middle-income countries, particularly in sub-Saharan Africa and South Asia, where undernutrition continues to hinder progress toward Sustainable Development Goals (SDGs) 2 and 3 [3,4]. Therefore, effective management requires not only caloric supplementation but also functional nutritional strategies that support recovery and long-term sustainability [5]. A growing body of evidence links undernutrition with gut dysfunction, including impaired mucosal barrier function, dysbiosis, and small intestinal bacterial overgrowth (SIBO) [6,7,8]. Chronic undernutrition weakens gut motility, mucosal immunity, and microbiota homeostasis, creating conditions in the small intestine that favor excessive bacterial proliferation [9,10,11]. Nutrient deficiencies further damage intestinal wall integrity and reduce immune mediators, such as secretory IgA, as well as digestive enzyme activity, allowing bacteria to multiply [12]. As SIBO develops, bacteria compete with the host for nutrients, increase intestinal permeability, and promote localized inflammation, thereby worsening macronutrient and micronutrient malabsorption and perpetuating the cycle of undernutrition [13,14,15]. This gut–nutrition–infection cycle illustrates why interventions should aim not only to restore body weight but also to improve intestinal health [16,17]. In this review, SIBO refers specifically to excessive bacterial colonization of the small intestine, as diagnosed using accepted diagnostic methods such as glucose or lactulose breath testing or culture-based small-bowel aspirate assessment. Therefore, SIBO is not used interchangeably with dysbiosis, microbiota immaturity, or general microbial imbalance.

Plant-derived hydrolysates and bioactive peptides have emerged as promising functional compounds for addressing this cycle because they may support tissue repair, immune responses, mucosal barrier integrity, and gut microbiota balance. These interventions may strengthen the intestinal barrier by supporting multiprotein junctional complexes, including occludin and claudins, while also encouraging commensal taxa, such as *Bifidobacterium* and *Lactobacillus*, and reducing the relative abundance of pathobionts, i.e., commensal organisms that become harmful when the microbial community is disturbed [18,19]. They may also exert anti-inflammatory effects by inhibiting NF-κB signaling and reducing pro-inflammatory cytokines, including IL-6 and TNF-α [20]. In addition, short di- and tripeptides may be absorbed through transport systems such as PEPT1, a process that may be particularly important during undernutrition [21]. Plant protein sources such as soybeans, chickpeas, lentils, and rice are more durable, affordable, culturally familiar in food-insecure regions, and environmentally favorable than animal-based proteins, thereby supporting SDGs 12 and 13 [22,23,24].

Despite their potential, bioactive peptides face limitations related to rapid degradation, reduced biological effectiveness, and poor bioavailability. Nanotechnology-based delivery systems, including protein nanoparticles, nanoencapsulation, and polymeric carriers, are being explored to protect peptides from degradation and improve intestinal absorption [25,26,27]. These approaches may enhance peptide functionality and help integrate nutritional and therapeutic strategies for vulnerable populations [28,29].

Undernutrition can impair intestinal epithelial renewal, gut motility, digestive enzyme secretion, and mucosal immune defense, including secretory IgA production. These changes may weaken the intestinal barrier and create conditions that favor bacterial overgrowth in the small intestine. Once SIBO develops, excessive bacteria may compete with the host for nutrients, increase mucosal inflammation, alter bile-acid metabolism, and aggravate intestinal permeability. This establishes a reinforcing cycle in which undernutrition promotes gut dysfunction, while SIBO further worsens nutrient malabsorption. This review examines mechanistic evidence on bioactive peptides, where available, in relation to intestinal barrier protection, inflammatory signaling, oxidative stress regulation, nutrient absorption, and microbiota modulation in undernutrition. Because direct peptide-intervention studies using clinically defined SIBO endpoints are limited in the reviewed literature, SIBO is treated as a plausible but unvalidated target rather than an established therapeutic outcome.

## 2. Materials and Methods

### 2.1. Literature Search Strategy

In January 2026, a literature search was conducted for this scoping review. Five electronic databases were searched: Scopus, Google Scholar, Web of Science, PubMed, and the Cochrane Library. Studies published between January 2010 to August 2026 were considered. The core search string included the following terms: (“bioactive peptide” OR “plant protein peptide” OR “protein hydrolysate” OR “antimicrobial peptide”) AND (“gut microbiota” OR microbiome OR “small intestinal bacterial overgrowth” OR SIBO OR “intestinal barrier” OR “gut permeability” OR “tight junction”) AND (“undernutrition” OR wasting OR “nutrient absorption”). Searches were adapted for each database using MeSH or Emtree terms where applicable. Filters were applied for publication date and English language, and searches were limited to peer-reviewed articles.

### 2.2. Study Selection

All retrieved records were imported into EndNote X9, and duplicates were removed. Screening discrepancies were resolved through consensus or by consulting a third assessor. This review evaluated the benefits of bioactive peptides derived from plant proteins for improving gut health in childhood undernutrition, with a focus on their protective effects on the intestinal barrier, regulation of oxidative stress, and mechanisms relevant to small intestinal bacterial overgrowth. Clinical and preclinical studies were considered eligible if they addressed plant-derived peptides and their interactions with gut microbiota, including *Bifidobacterium*, *Lactobacillus*, and pathobiont suppression, gut barrier function, including tight-junction proteins such as occludin, claudins, and ZO-1, inflammation and immune responses, including NF-κB, IL-6, and TNF-α, nutrient absorption, including PEPT1 transporters and amino acid uptake, and growth-promotion indicators, including mid-upper arm circumference, weight gain, and height-for-age.

### 2.3. Data Extraction

Eligible studies included in vitro, animal, and human studies evaluating plant-derived peptides or plant-protein hydrolysates in relation to gut barrier integrity, microbiota modulation, SIBO-related mechanisms, inflammation, oxidative stress, or nutrient absorption. Reviews, editorials, letters, studies on non-protein bioactive compounds, studies on peptides not derived from plants, and studies without relevant gut-health outcomes were excluded.

A total of 1300 studies were identified from Scopus, Google Scholar, PubMed, Web of Science, and the Cochrane Library. Of these, 564 were removed before screening because they were duplicates, were flagged as ineligible by automation tools, or were removed for other reasons, leaving 736 records for title and abstract screening. Following screening, 242 studies were excluded, and 494 studies were sought for full-text retrieval. Of these, 181 reports were not retrieved or were excluded because they were review, papers, letters, or conference papers, leaving 313 reports assessed for eligibility. Of these, an additional 181 were excluded because of inadequate study design, insufficient data, unsuitable study type, or because they assessed non-protein bioactive complexes. A final total of 132 studies met all inclusion criteria and were included in this scoping review.

For human intervention studies, clinical evidence was considered valid only when outcomes were compared between a bioactive-peptide intervention group and a relevant control group, with baseline differences accounted for through change-from-baseline analysis, adjusted models, or equivalent comparative reporting. Studies reporting only within-group baseline-to-endpoint changes were not treated as evidence of clinical efficacy and were summarized as preliminary or exploratory findings.

**Figure 1:**
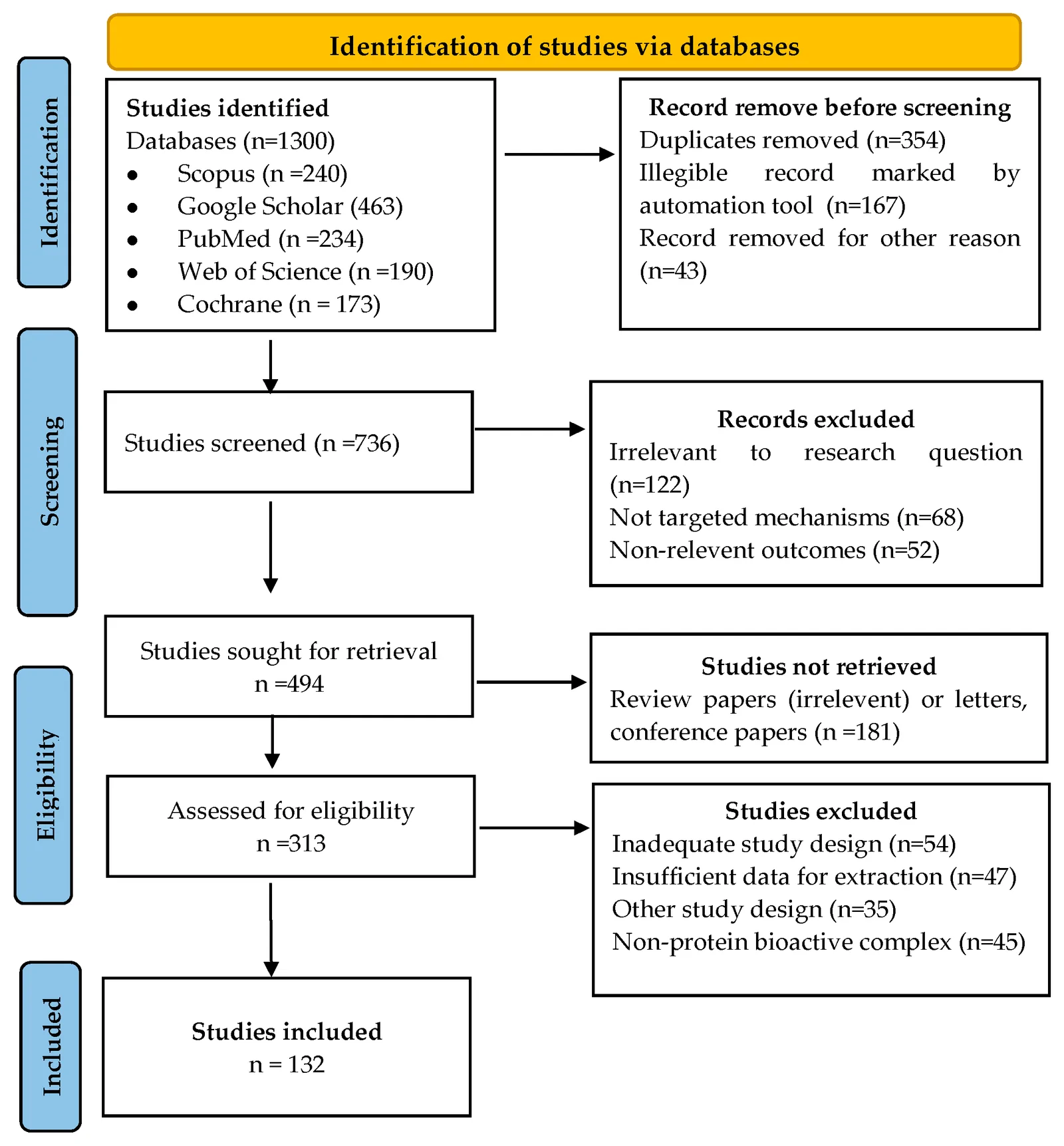
Literature search flow diagram showing the study selection process based on the inclusion and exclusion criteria.

For this scoping review, the inclusion criteria comprised studies on plant-derived peptides or plant-protein hydrolysates, gut barrier function, oxidative stress, inflammation, nutrient absorption, microbiota modulation, undernutrition, or clinically defined SIBO. The exclusion criteria comprised studies on non-peptide phytochemicals, unless used only for contextual comparison; studies without gut-relevant endpoints; and studies using dysbiosis or microbiota immaturity as a substitute for SIBO without clinical testing. The study selection process is summarized in the schematic flow diagram shown in Figure 1.

### 2.4. Evidence Categories

Interventions of very different biological specificity are frequently grouped together in this literature, resulting in effects observed for a complex food matrix being attributed to peptides that were never isolated. To prevent this, every study discussed in this review is assigned to one of five categories, and the category is stated wherever the study is cited:

(i) Identified peptide: A chemically defined sequence, synthesized or purified to a stated purity, administered as the intervention.
(ii) Peptide fraction: A hydrolysate separated by molecular weight or chromatography, in which the active fraction is defined but the constituent sequences are not.
(iii) Protein hydrolysate: An enzymatic digest administered as a whole, with no isolation of the active component.
(iv) Intact protein or protein-rich food: A protein source administered without hydrolysis.
(v) Complex food matrix or non-peptide preparation: Microbiota-directed complementary foods, ready-to-use therapeutic foods, bioactive glycans, and comparable formulations, in which any peptide contribution is neither isolated nor quantified.

Effects observed in categories (iv) and (v) are not attributed to bioactive peptides at any point in this review. Where a study in these categories is discussed, it is presented as describing the clinical or ecological context in which peptide-based approaches would operate, and the distinction is stated explicitly in the surrounding text.

### 2.5. Basis for the Evidence-Strength Labels

Evidence-strength labels are assigned using the following rubric, applied to the body of evidence supporting each mechanism rather than to any single study. High: At least one randomized controlled trial in a population with childhood undernutrition, with a defined comparator and the mechanism-relevant outcome measured directly. Moderate: Consistent findings across two or more independent in vivo studies using a defined intervention and comparator, with the mechanism measured directly rather than inferred, supported by at least one study in a human-derived intestinal model. Low–Moderate: In vivo evidence from a single study, or in vitro evidence only in which the mechanism itself was measured directly. Low: Mechanism inferred from surrogate endpoints, or evidence drawn only from chemical assays or from studies in which no peptide fraction was isolated. Insufficient: No included study measured the outcome. No formal risk-of-bias instrument was applied, as this scoping review does not make formal risk-of-bias claims. This is noted as a limitation in Section 6.

## 3. Plant-Derived Peptides and Their Extraction Methods

Plant proteins are increasingly recognized as important sources of bioactive peptides with diverse biological functions relevant to human health and the development of functional foods. This section examines the extraction of plant-derived peptides from soybeans, chickpeas, peas, rice bran, quinoa, sesame seeds, corn, mung beans, lupin, oats, rapeseed, and Bambara groundnut using enzymatic hydrolysis and other advanced methods.

For example, soybeans have a high protein content and a balanced amino acid profile. Enzymatic hydrolysis of soybean proteins produces short-chain, peptide-rich hydrolysates with potent antioxidant effects. In particular, pepsin hydrolysates demonstrate increased antioxidant activity and protection against lipid peroxidation [30]. Peptide fractions derived from the pepsin-induced breakdown of soy protein isolate show enhanced antioxidant and anti-inflammatory properties, along with improved nutritional functionality [31].

Proteins from legumes, such as chickpeas, also provide potentially therapeutic peptides. After protein extraction and ficin-catalyzed hydrolysis, liquid chromatography–tandem mass spectrometry (LC-MS/MS) analysis has shown that germinated chickpea protein hydrolysates possess antidiabetic properties. These hydrolysates can regulate dipeptidyl peptidase-IV (DPP-IV) activity and decrease glucose uptake in intestinal cell models [32]. Further studies have demonstrated the antihypertensive effects of chickpea protein hydrolysates produced by Alcalase hydrolysis in hypertensive rats, mediated through the angiotensin-converting enzyme (ACE)2/Mas1 pathway [33].

Pea protein is another valuable source of functional peptides. Enzymatic hydrolysis using Alcalase, followed by molecular-weight fractionation, produces low-molecular-weight peptides (<3 kDa) with strong antioxidant and ACE-inhibitory activities [34]. Controlled enzymatic hydrolysis of pea protein concentrates has also been shown to reduce lipid accumulation and improve stress resistance in the model organism *Caenorhabditis elegans*, suggesting potential metabolic health benefits [35].

Optimizing the alkaline hydrolysis of mung bean protein hydrolysates improves their functional properties, making them suitable for incorporation into peptide-based functional food systems [36]. Hydrolysates of albumin from *Lupinus mutabilis* tarwi digested using *Micrococcus* sp. protease have generated multifunctional peptides with antioxidant, antihypertensive, and antidiabetic properties [37]. Advanced processing technologies have greatly enhanced the extraction and bioactivity of plant proteins. For example, ultrasound-assisted enzymatic hydrolysis of oat proteins improves peptide extraction and alters protein structure. Peptides prepared from oats have been found to exert neuroprotective and antioxidant effects in PC12 neuronal cells and zebrafish models [38].

Cereal and pseudocereal proteins have also been extensively studied for peptide generation. Rice bran protein hydrolysates produced through enzymatic hydrolysis using *Bacillus licheniformis* protease or α-chymotrypsin exhibit significant antioxidant properties, such as metal-chelating capacity and inhibition of lipid peroxidation, especially in low-molecular-weight fractions [39]. Quinoa protein hydrolysates produced using various proteases, particularly Alcalase, are rich in low-molecular-weight peptides that exhibit cytoprotective and antioxidant properties in cell models exposed to oxidative stress [40]. Furthermore, research on corn gluten meal has identified it as a source of antioxidant cyclic peptides generated through a bioinformatics-driven enzymatic hydrolysis process using papain and subtilisin, resulting in peptides with potent radical-scavenging capabilities [41]. These findings highlight the growing importance of computational tools and enzyme-selection strategies in enhancing peptide production from plant-based proteins.

Byproducts from oilseeds and legumes hold significant promise for peptide extraction and valorization. Hydrolysis of sesame protein byproducts with Alcalase has produced peptide-rich fractions exhibiting a range of biological activities, including antioxidant, antihyperglycemic, anti-obesity, and antidiabetic effects in vitro, suggesting potential relevance to gut-related functions [42]. Peptides from Bambara groundnut, produced through Flavourzyme hydrolysis followed by membrane fractionation and LC-MS/MS analysis, demonstrated dual inhibitory effects on ACE and DPP-IV enzymes, suggesting their potential for preventing cardiometabolic diseases [43]. Furthermore, rapeseed albumin hydrolysates generated using the primary cell-envelope serine protease PrtS from *Streptococcus thermophilus* have demonstrated antioxidant and anti-inflammatory effects while maintaining beneficial functional characteristics for food applications [44].

Preclinical studies were used to evaluate physiological plausibility, and human studies were considered separately to avoid overstating clinical translation. Mechanistic findings were not interpreted as clinical efficacy unless supported by controlled human intervention data with appropriate comparisons against baseline values and control groups. The plant protein sources, extraction methods, structural characteristics, evidence models, and reported bioactivities of plant-derived protein hydrolysates and peptides are documented in Table 1.

## 4. Mechanisms of Action of Plant Protein Hydrolysates and Peptides

### 4.1. Gut Barrier Integrity

The intestinal barrier comprises mechanical, chemical, immunological, and biological components that together maintain intestinal homeostasis and limit exposure to luminal pathogens [54,55,56]. The mechanical barrier is formed by the mucosal epithelium, lamina propria, and muscularis mucosae [57], and its selective permeability depends on intestinal epithelial cells joined by apical junctional complexes comprising adherens junctions, tight junctions (TJs) and desmosomes [58]. Whereas adherens junctions and desmosomes principally maintain cell–cell adhesion, TJs regulate paracellular transport. TJs are formed by transmembrane components, including claudins, occludin, and junctional adhesion molecules (JAMs), which are anchored to the actin cytoskeleton by zonula occludens scaffolding proteins ZO-1, ZO-2, and ZO-3. Together, these restrict paracellular flux and preserve apical–basal cell polarity [57,59].

Within this complex, pore-forming claudins act as paracellular ion channels, whereas barrier-forming claudins restrict paracellular flux [60]. Occludin contributes to TJ assembly through its MARVEL motif and is supported by ZO-1, a membrane-associated guanylate kinase-like scaffold that links the junction to F-actin [61]. Barrier function is further tuned post-translationally: claudin-1 is phosphorylated by atypical protein kinase C, protein kinase A, and mitogen-activated protein kinases, and phosphorylation generally promotes claudin assembly into TJs, whereas phosphorylation of occludin enhances sealing [53]. Increased expression of barrier-forming claudins, occludin, and JAMs is accordingly associated with reduced paracellular permeability and improved barrier function [62].

**Figure 2:**
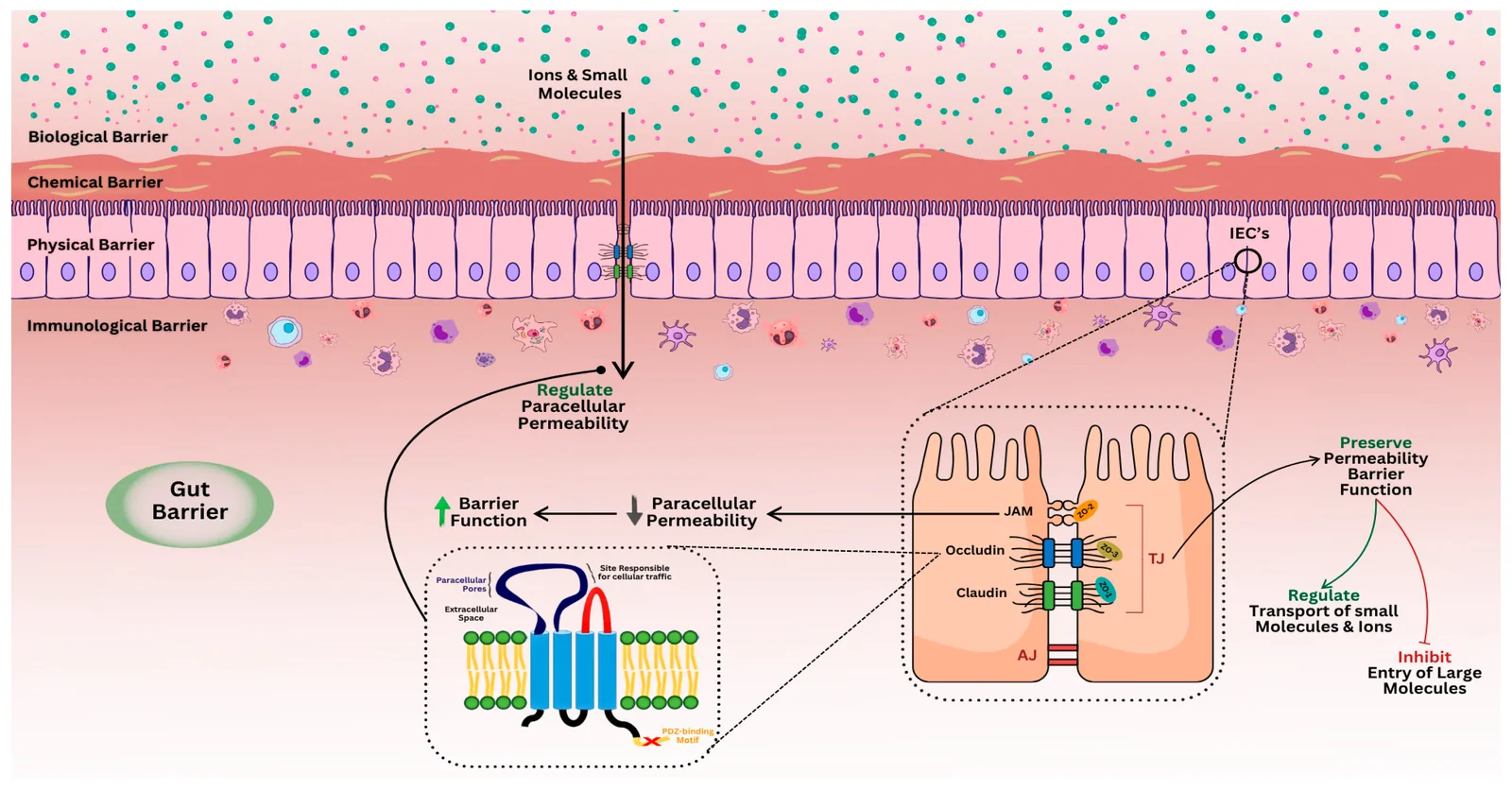
Schematic representation of the intestinal barrier and regulation of epithelial permeability. The gut barrier consists of biological, chemical, physical, and immunological components that maintain intestinal homeostasis by controlling the movement of ions, small molecules, and larger substances. Tight junctions (TJs), including junctional adhesion molecules (JAMs), claudins, and occludin, regulate paracellular permeability and preserve epithelial barrier function [45,57,58,59,60,61].

The mechanistic evidence is broadly consistent across several pathways. Plant protein-derived peptides may enhance intestinal barrier function by stimulating the expression of TJ proteins, thereby reducing paracellular permeability to pathogens and microbial antigens. This aligns with evidence identifying occludin, claudins, and ZO-1 as essential components of the intestinal barrier that regulate selective permeability and prevent the systemic translocation of luminal antigens, bacteria, and microbial toxins [54,57,63,64,65]. The structural and functional regulation of the intestinal epithelial barrier and tight junction complexes by plant peptides is shown in Figure 2.

### 4.2. Role of Plant Protein Hydrolysates and Peptides in Gut Barrier Integrity

Plant-derived bioactive components exhibit multiple bioactivities, including the ability to modulate the gut microbiota and enhance gut barrier function, which may help prevent chronic diseases [56]. Bioactive peptides support physiological functions and contribute to gastrointestinal health by promoting barrier function, immune responses, and gut microbiota balance. Plant peptides can be derived from leaves, seeds, and fruits, with seeds being among the most cost-effective protein sources [66].

Across the studies retained in Table 1, increased expression of tight-junction proteins is the most consistently reported effect of plant-derived protein hydrolysates and peptides on the intestinal barrier. Oat protein hydrolysate at 500 mg/kg/day increased colonic occludin and claudin-1 protein levels and ZO-1 and occludin mRNA, alongside increased MUC2 expression, goblet cell numbers, and mucus secretion [45]. Rice protein hydrolysate fraction at 100 and 500 mg/kg increased colonic ZO-1, occludin, and claudin-1 [46]. Wheat protein hydrolysate at 200 and 500 mg/kg restored colonic ZO-1, occludin, and claudin-1 at both the mRNA and protein levels and recovered goblet cell number and MUC2 expression [48]. Foxtail millet protein hydrolysate at 400 mg/kg/day increased colonic ZO-1 and occludin at the mRNA and protein levels and restored transepithelial electrical resistance in LPS-challenged Caco-2 monolayers, but had no significant effect on claudin-1, claudin-3, MUC2, or MUC3 [47]. Low-dose selenium-enriched soybean peptide fraction increased colonic ZO-1 and occludin, whereas the high dose restored occludin only [50], and corn gluten meal hydrolysate reduced serum diamine oxidase, an indirect marker of intestinal permeability, without measuring junctional proteins directly [49].

Three features of this evidence qualify its interpretation. The response is not uniform across junctional proteins: ZO-1 and occludin change most consistently, whereas claudin responses were variable and, in one of the four studies that measured them, absent, which indicates that these interventions do not act on the junction as a single unit. Only two studies measured a functional permeability endpoint alongside protein expression, so in most cases, the inference from increased protein abundance to improved barrier function remains indirect. Finally, all of these observations derive from chemically induced colitis rather than from undernutrition, and no included study measured intestinal permeability in an undernourished animal or human population.

**Figure 3:**
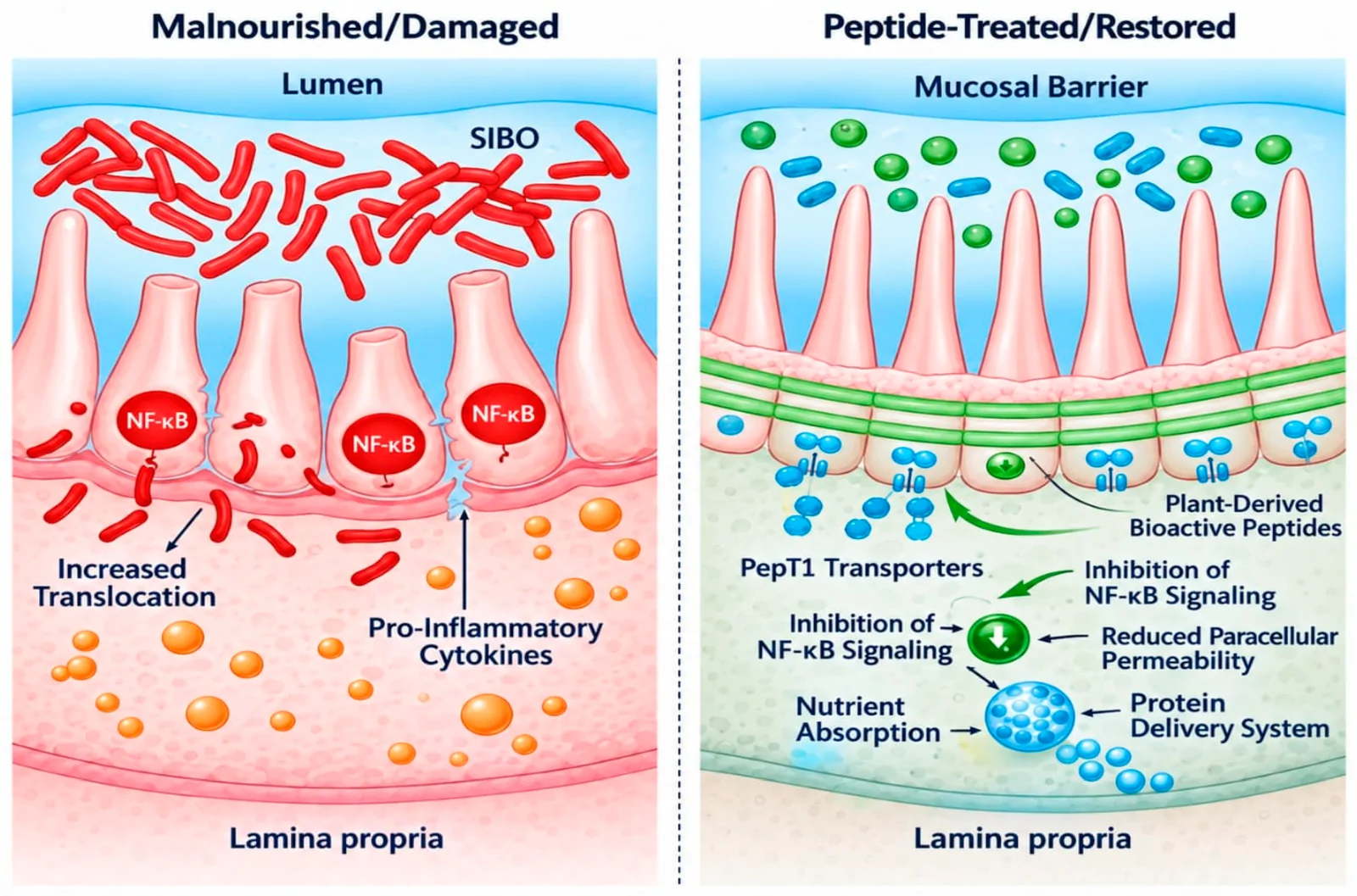
Proposed mechanisms by which plant-derived hydrolysates and bioactive peptides may support the restoration of gut health. Damaged mucosa shows microbial imbalance, increased microbial translocation, activation of nuclear factor kappa B (NF-κB) signaling, and release of pro-inflammatory cytokines. Transportable di- and tripeptides may undergo peptide transporter 1 (PEPT1)-mediated uptake, whereas enhancement of PEPT1 expression or transport activity was not measured in the included studies. Peptide treatment may improve nutrient absorption indirectly, reduce paracellular permeability, and strengthen the mucosal barrier. These combined effects may suppress inflammation, support epithelial repair, and promote intestinal homeostasis [54,55,56,57,58,59,60,61,62]. The damaged state represents barrier disruption and microbial translocation. It does not represent clinically defined small intestinal bacterial overgrowth, which was not measured in any study.

Soy proteins are a major source of plant-derived bioactive peptides, with glycinin and β-conglycinin accounting for more than 85% of soy proteins [67]. The wider literature attributes a broad range of activities to plant protein hydrolysates: radical scavenging in cell-free systems, antihypertensive and immunomodulatory activity for wheat gluten hydrolysates [68], and antioxidant, hypocholesterolemic, ACE-inhibitory, and metal-chelating activity for chickpea and pumpkin seed hydrolysates [69,70]. Most of these activities were measured outside the intestine and are not gut-relevant endpoints. They are noted here for context and are not carried forward into the mechanisms discussed below. Figure 3 illustrates how plant-derived bioactive peptides may contribute to the restoration of gut health.

### 4.3. Effects on the Gut Microbiota

A distinction should be drawn between two categories of plant peptides that are frequently conflated. The first comprises classical antimicrobial peptides, which are gene-encoded, cysteine-rich defense molecules such as defensins, thionins, lipid-transfer proteins, and cyclotides, which act directly on microbial membranes and for which minimum inhibitory concentration values are routinely reported [69,71,72]. The second comprises the food-derived plant protein hydrolysates and enzymatically released peptides that constitute the evidence base of this review. These are not gene-encoded defense molecules, and none of the studies in Table 1 measured direct antibacterial activity. No minimum inhibitory concentration, time-kill curve, or zone-of-inhibition assay was reported. Statements that food-derived plant protein hydrolysates or peptides suppress pathogens therefore rest on compositional shifts in the intestinal microbiota rather than on demonstrated bactericidal action.

What these studies do show is selective modulation of community composition in vivo. Oat protein hydrolysate at 500 mg/kg/day increased *Coriobacteriaceae* and *Pediococcus* and decreased *Bacteroides* in DSS-treated mice [45]. Wheat protein hydrolysate at 500 mg/kg increased *Desulfovibrio* and *Odoribacter*, reduced *Bacteroides* and *Escherichia-Shigella*, and shifted the overall community toward the untreated control profile [48]. In both studies, these shifts were accompanied by increased cecal acetic, propionic, and butyric acids, offering a plausible indirect route to colonization resistance, since short-chain fatty acids lower luminal pH and inhibit *Enterobacteriaceae*. However, neither study isolated the responsible peptide fraction, tested it against the affected taxa in pure culture, or established that the microbial change preceded rather than followed mucosal healing.

In the studies included here, the microbiota was characterized in cecal contents, and the wider literature on peptide–microbiota interactions relies predominantly on fecal sampling. Neither compartment represents the proximal small intestine, where bacterial density is normally several orders of magnitude lower and where small intestinal bacterial overgrowth occurs. Fecal and cecal communities are dominated by colonic anaerobes and are poorly correlated with duodenal or jejunal bacterial load, so an increase in fecal *Bifidobacterium* or a decrease in fecal *Bacteroides* cannot be read as evidence that small-bowel overgrowth has been reduced. Direct assessment requires small-bowel aspirate culture or, indirectly, breath testing, and neither was performed in any study reviewed here. Distal sampling therefore cannot substitute for small-bowel measurement, and the microbiota findings summarized above should be interpreted as colonic outcomes.

Outside the studies retained in Table 1, a larger body of work describes dietary peptides as substrates and signals for the intestinal community rather than as antibacterial agents. Peptides reaching the distal gut may act as a nitrogen source supporting bacterial growth and have been proposed to assist the movement of polypeptides to particular cell sites [73]. Indigestible proteins and hydrolysates, including hydrolyzed soy protein, shift community composition in a manner similar to unabsorbed sugars, resistant starch, and fiber do [74]. Pea protein extract increases *Bifidobacterium* and *Lactobacillus* and raises intestinal short-chain fatty acid concentrations [75]. Soy protein and fermented soy foods have been reported to reduce the *Firmicutes-to-Bacteroidetes* ratio in rat cecum, an effect attributed in part to isoflavones rather than to peptides [76], and soybean intake has been associated with increased *Escherichia* and *Propionibacterium* [77]. Interventions with lactic acid bacteria and *Bifidobacterium* reduce *Firmicutes* and increase *Actinobacteriota* at the phylum level, with short-chain fatty acid production as one proposed route [78].

Growth inhibition of *Escherichia coli* and *Clostridium perfringens* by protein hydrolysates has been reported in vitro [79]. That work used different preparations from those in Table 1, the tested concentrations had no defined relationship to luminal exposure after ingestion, and none of the included studies repeated the measurement. Taken together, this literature supports a nutritional and ecological role for dietary peptides in the colon. It does not establish bactericidal action in vivo, and it does not address bacterial density in the small intestine.

### 4.4. Regulation of the Gut–Brain Axis: Scope and Limits of the Present Evidence

Environmental enteric dysfunction manifests as intestinal leakiness and heightened permeability, gut inflammation, dysbiosis, and bacterial translocation [7], and such disturbances generate signals that reach the central nervous system through vagal, immune, and endocrine routes, as well as through microbial metabolites, including short-chain fatty acids [80,81,82]. Within the assembled evidence base, however, a single included study measured a neurobehavioral endpoint: wheat protein hydrolysate administered at 200 and 500 mg/kg reversed the reductions in total distance traveled, distance moved in the central zone, time spent in the central zone, and number of central-zone entries observed in the open field test in mice with dextran sulfate sodium-induced colitis, alongside restored colonic tight-junction proteins and reduced colonic and serum IL-1β, IL-6, and TNF-α [48]. Beyond this observation, the evidence base does not extend to neurotransmitter concentrations, vagal signaling, or cognitive performance in undernourished animal or human populations; for plant-derived peptides, these remain unmeasured, and no study has yet examined such peptides in a neurodevelopmental disorder. Such measurements would be required to convert a plausible link into a demonstrated one, and gut–brain signaling is accordingly treated in this review as a priority for future work rather than as an established action of the peptides discussed.

### 4.5. Regulation of Oxidative Stress Through the Keap1–Nrf2–ARE Pathway

Oxidative stress in undernutrition-related gut dysfunction may arise from excessive reactive oxygen species, impaired epithelial defense, inflammation, and microbial imbalance [7]. The Keap1–Nrf2–ARE pathway is a central antioxidant-response system: under oxidative stress, Nrf2 dissociates from Keap1, translocates to the nucleus, binds antioxidant response elements, and promotes transcription of cytoprotective enzymes, including heme oxygenase-1, NAD(P)H quinone oxidoreductase 1, superoxide dismutase, catalase, and glutathione-related enzymes [19]. Evidence that plant-derived peptides engage this pathway, rather than merely scavenging radicals in cell-free chemical assays, is confined to studies in which Nrf2 or its downstream targets were measured directly; of the studies retained in Table 1, five report intestinal redox markers, but only three measured the pathway itself. Rice protein hydrolysate fraction at 100 and 500 mg/kg body weight, administered by gavage for 14 days, increased nuclear Nrf2 with reduced cytoplasmic Keap1 and induced HO-1, NQO1, and Gclc mRNA in colitis mice [46]. Oat protein hydrolysate at 500 mg/kg/day for 15 days reduced colonic malondialdehyde and increased superoxide dismutase and total antioxidant capacity in vivo, while the oat protein hydrolysate and the synthetic sequence WGVGVRAERDA at 50 µg/mL reduced Keap1 and promoted Nrf2 nuclear translocation in Caco-2 cells, as assessed by immunofluorescence [45]. Wheat protein hydrolysate at 50 µg/mL reduced Keap1 protein and increased Nrf2 protein with enhanced nuclear localization in Caco-2 cells, and the same direction of effect was confirmed by Western blotting in colon tissue from mice receiving 200 or 500 mg/kg by gavage [48]. By contrast, corn gluten meal hydrolysate at 100, 300, and 500 mg/kg/day increased colonic glutathione peroxidase and superoxide dismutase in a dose-dependent manner but examined Keap1 only by molecular docking [49], and selenium-enriched soybean peptide fraction at 15 and 30 µg Se/kg body weight/day for 23 days restored colonic glutathione peroxidase and total superoxide dismutase without measuring Nrf2 [50].

### 4.6. Anti-Inflammatory Effects of Plant-Derived Peptides

Peptides from various sources have been shown to effectively inhibit inflammation in cell culture studies. Dipeptides derived from yeast extracts and edible beans have demonstrated anti-inflammatory activity in Caco-2 cells. Specifically, they were reported to reduce the secretion of the pro-inflammatory cytokines IL-8, IL-6, and IL-1β while enhancing the expression of the anti-inflammatory cytokine IL-10 in TNF-α-induced cells [83].

Bioactive peptides play a significant role in modulating the immune response, particularly in inflammation and related diseases. These peptides possess immunomodulatory properties and interact with immune cells and signaling pathways to modulate specific immune mediators. Some bioactive peptides have direct and indirect anti-inflammatory effects, reducing the production of pro-inflammatory cytokines and aiding in the management of chronic inflammatory conditions [84,85,86]. Additionally, bioactive peptides impact gut health and the gut microbiota, indirectly influencing the immune system. A balanced gut microbiota supports a well-regulated immune response, whereas strengthened gut immunity helps defend against infections, reduce inflammation, and promote overall gut health. These peptides, with their diverse functionalities, exhibit prebiotic-like effects by selectively promoting the growth of beneficial bacteria and limiting the expansion of pathobionts, thus supporting gut health and immune function [87].

In one study assessing the antioxidant and anti-inflammatory protective effects of bioactive peptides, the walnut protein hydrolysate-derived peptide (WEKPPVSH) was studied. It was found that this peptide significantly decreased nitric oxide (NO) and reactive oxygen species (ROS) production in a dose-dependent manner and upregulated superoxide dismutase and catalase activities. WEKPPVSH also significantly reduced the secretion of TNF-α, IL-1β, and IL-6 [88]. Eighteen peptides were identified in walnut protein hydrolysate that demonstrated anti-inflammatory effects in lipopolysaccharide (LPS)-activated murine microglial BV-2 cells. This study revealed that Trp, Gly, and Leu residues within the peptides may contribute to their anti-inflammatory effects. Specifically, the peptide WEKPPVSH from walnut protein hydrolysate exhibited anti-inflammatory effects in lipopolysaccharide (LPS)-activated BV-2 microglia by modulating oxidative stress [88].

The plant-derived mechanistic evidence comes from the studies retained in Table 1. Foxtail millet protein hydrolysate at 400 mg/kg/day reduced colonic TNF-α, IL-6, IL-17, and IL-1β, inhibited NF-κB p65 phosphorylation, reduced the colonic CD4^+^ IL-17^+^ Th17 population, and lowered NLRP3, ASC, and cleaved caspase-1; the effects on p65 phosphorylation and inflammasome components were reproduced in LPS-challenged Caco-2 monolayers at 200 µg/mL, where the hydrolysate also restored transepithelial electrical resistance [47]. Wheat protein hydrolysate at 200 and 500 mg/kg reduced colonic and serum IL-1β, IL-6, and TNF-α at both the mRNA and protein levels in mice with dextran sulfate sodium-induced colitis [48]. Comparable reductions in colonic pro-inflammatory cytokines were reported for oat protein hydrolysate at 500 mg/kg/day [45] and for corn gluten meal hydrolysate at 100, 300, and 500 mg/kg/day [49], and the soy glycinin-derived tripeptide VPY reduced TNF-α-induced IL-8 secretion in intestinal epithelial monolayers [51].

Two limitations apply to this evidence. First, in the millet, oat, and corn studies, the intervention was a whole hydrolysate (category iii, Section 2.4), so the effect cannot be attributed to any individual sequence. Only the soy and wheat germ studies tested defined sequences against an inflammatory endpoint. Second, all of these models are chemically induced colitis rather than undernutrition. The dextran sulfate sodium colitis model reproduces barrier disruption and mucosal inflammation, but does not reproduce the nutrient deficiency, growth faltering, or enteropathy that characterize childhood undernutrition, and the extrapolation from one to the other is an interpretive assumption of this review rather than a finding of the primary literature.

IL-1β, TNF-α, and IL-6 are major mediators of inflammation in conditions such as inflammatory bowel disease (IBD), obesity, and similar inflammation-related diseases [89]. When assessing the anti-inflammatory effects of plant-derived bioactive peptides, evidence suggests that they may influence key phosphorylation events in the NF-κB pathway and modulate phosphorylation cascades, ultimately reducing inflammation. The p50 and p65 subunits serve as the primary transcription factors in the NF-κB pathway. Numerous studies indicate that plant-derived bioactive peptides sourced from cereals, nuts, and other plants can inhibit p65 translocation, potentially suppressing the release of inflammatory factors [90]. In another study, bean peptides were found to modulate the NF-κB signaling pathway through transcriptomic analysis, resulting in reduced inflammation [91].

### 4.7. Effect of Peptides on Nutrient Absorption

L-type amino acid transporters (LATs), including LAT1/SLC7A5, primarily mediate the transport of neutral amino acids and should not be described as major transporters of intact peptides. By contrast, the intestinal uptake of di- and tripeptides is mediated mainly by proton-coupled oligopeptide transporters (POTs), particularly PEPT1/SLC15A1. Therefore, any discussion of peptide-enhanced nutrient absorption should focus on PEPT1/POT-mediated peptide transport, whereas LATs should be limited to free amino acid transport. In addition, ultra-short peptides may exert biological effects by influencing endocrine, nervous, and immune functions, partly through the regulation of gene expression and protein synthesis [92].

Two qualifications follow from the substrate specificity of PEPT1. First, PEPT1 accepts di- and tripeptides only. Peptides of four or more residues are not transported intact by this route [20]. Most of the sequences discussed in this review exceed that limit. WGVGVRAERDA is eleven residues, and GIIQPQQPAQLEVIR is fifteen. Therefore, their intestinal effects cannot be attributed to PEPT1-mediated uptake. Where such peptides act, they are more likely to do so at the apical membrane, through receptor interactions, or via luminal signaling, rather than by intact transport. Whether they undergo further hydrolysis to transportable fragments was not examined in any included study. Second, no study included in this review measured PEPT1 expression, transport activity, or peptide flux following administration of a plant protein hydrolysate. The proposition that hydrolysate ingestion enhances PEPT1-mediated absorption is therefore inferred from the known physiology of the transporter rather than observed.

Mineral-chelating peptides have been proposed as a route to improving mineral status, since peptide–metal complexes can form stable structures that limit interaction with other food components and reduce the pro-oxidant activity of iron [93]. The best-characterized examples are not plant-derived: casein phosphopeptide–calcium chelate was more bioavailable than calcium chloride or calcium L-aspartate and increased TRPV6 and TRPV5 expression, indicating improved cellular calcium uptake [55]. No study included in this review measured calcium, iron, or zinc bioavailability following administration of a plant protein hydrolysate. Mineral chelation is therefore presented here as a mechanism established for other protein sources and untested for the intervention class under review.

SCFAs are short-chain fatty acids, including acetate, propionate, and butyrate, and are end products of bacterial fermentation. These metabolites act as signaling molecules that regulate various transcription factors involved in energy balance. SCFAs can influence appetite and food intake by affecting brain activity through direct or indirect mechanisms [94]. SCFAs can bind to receptors on enteroendocrine cells, prompting the release of hormones related to appetite into the systemic circulation, thereby modifying energy balance and food intake. For example, acetate can directly suppress appetite through central hypothalamic mechanisms [95,96]. Mitev and Taleski reported that gut microbiota affects energy balance and the development of obesity by inhibiting insulin-mediated fat accumulation through SCFA receptors, primarily acetate receptors [95].

## 5. Potential Role of Plant-Derived Peptides in Gut Barrier and Microbiota Recovery in Childhood Undernutrition

### 5.1. Restoration of Gut Barrier Function

Undernutrition can compromise the structural and functional integrity of the intestinal epithelium, thereby increasing intestinal permeability, which is colloquially referred to as “leaky gut”. This impairment of the mucosal barrier allows the passage of intraluminal bacteria and toxins into the circulation [54]. Impaired barrier function, together with altered motility and mucosal defense, may facilitate bacterial colonization within the small intestine, thereby contributing to the development or persistence of SIBO [19]. Plant-derived protein hydrolysates and peptides have been reported to attenuate this barrier dysfunction in experimental colitis models, increasing mucosal TJ protein expression, including occludin, claudin-1, and ZO-1 [45,46,48]. TJs are critical determinants of the selective permeability of the intestinal lining. Strengthening these junctions may reduce paracellular gaps between epithelial cells. These interventions may enhance the expression and polymerization of TJ proteins, thereby decreasing paracellular permeability and the translocation of luminal bacteria and antigens [64]. Restoration of mucosal barrier function reduces epithelial permeability and limits the opportunity for bacterial translocation. By strengthening tight-junction integrity and reducing inflammatory permeability, bioactive peptides may help create conditions less favorable to bacterial overgrowth; however, direct evidence that plant-derived peptides reduce clinically diagnosed SIBO is not yet available [97].

### 5.2. Microbiota Modulation and Colonization Resistance

Plant-derived protein hydrolysates and peptides may influence microbial ecology in the undernourished gut, but the underlying mechanism determines the strength of the claim that can be made. As set out in Section 4.3, no study included in this review measured direct antibacterial activity for a food-derived plant hydrolysate or peptide. The mechanism supported by the present evidence is modulation of community composition, not bacterial killing [98].

Two observations are relevant to colonization resistance. The oat and wheat protein hydrolysates shifted the cecal community toward the untreated control profile and increased cecal acetic, propionic, and butyric acids in mice with dextran sulfate sodium-induced colitis [45,48]. SCFAs lower luminal pH and restrict *Enterobacteriaceae*, which offers an indirect route by which hydrolysate or peptide feeding could narrow the niche available to pathobionts such as *Enterobacteriaceae* and *Clostridium*, taxa frequently enriched in SIBO [99]. Plant and dairy protein preparations have been reported to shift community composition toward *Bifidobacterium* and *Lactobacillus* [100], and soy protein and peptides have been associated with increased commensal populations and reduced Bacteroidetes [50]. Whether any of this alters bacterial density in the proximal small intestine, which is what defines SIBO, has not been tested. All available measurements are colonic.

**Figure 4:**
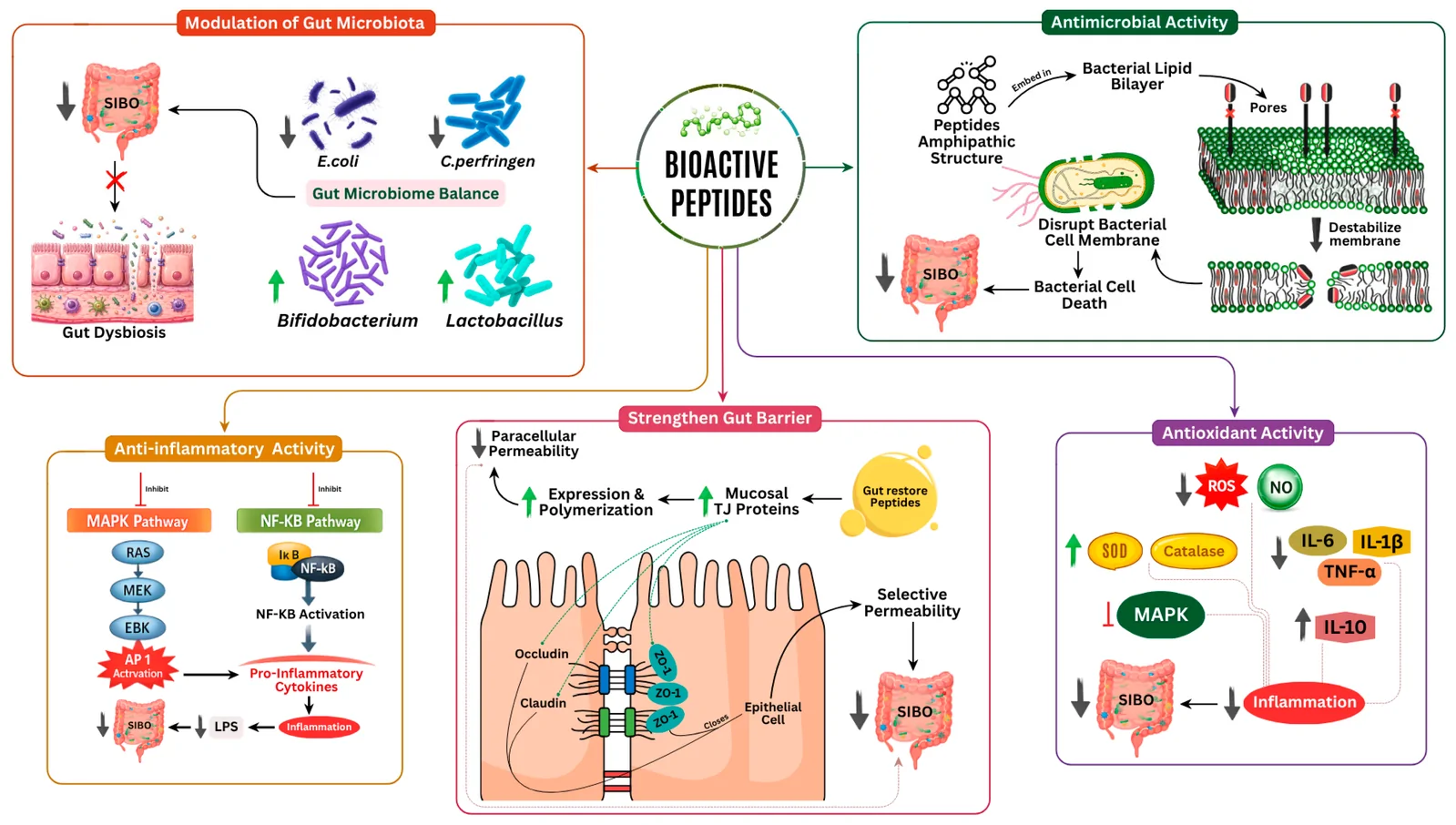
Proposed multifunctional mechanisms of bioactive peptides in improving gut health. Bioactive peptides may modulate the gut microbiota, shifting community composition away from pathobionts such as *Escherichia coli* (*E. coli*) and *Clostridium perfringens* (*C. perfringens*) and toward *Bifidobacterium* and *Lactobacillus.* They may also increase tight junction (TJ) protein expression, including zonula occludens-1 (ZO-1), zonula occludens-3 (ZO-3), occludin, and claudin, and decrease paracellular permeability. Bioactive peptides have been reported to inhibit mitogen-activated protein kinase (MAPK), nuclear factor-kappa B (NF-κB), activator protein-1 (AP-1), lipopolysaccharide (LPS)-mediated signaling, reactive oxygen species (ROS), nitric oxide (NO), interleukin-6 (IL-6), interleukin-1 beta (IL-1β) and tumor necrosis factor-α (TNF-α), and to stimulate antioxidant enzymes, including superoxide dismutase (SOD) and catalase, and the anti-inflammatory cytokine interleukin-10 (IL-10) [54,91,98,99,100,101,102,103,105,107].

Anti-inflammatory action may reinforce the same pattern. Inhibition of NF-κB signaling, blockade of p65 translocation, and reduced TNF-α, IL-8, IL-6, and IL-1β with increased IL-10 have been reported for plant peptides [91,101,102,103], and MAPK signaling has also been implicated [78,104]. Because mucosal inflammation increases permeability and alters the luminal environment, its suppression is plausibly relevant to the conditions under which overgrowth develops [87,105]. This is a mechanistic argument rather than a demonstrated effect on SIBO [106]. The mechanisms by which bioactive peptides modulate gut barrier function, inflammation, oxidative stress, and gut microbiota are shown in Figure 4.

### 5.3. Anti-Inflammatory Action

SIBO may be associated with low-grade chronic inflammation caused by bacterial toxins, such as LPS. Previous studies have demonstrated that bioactive peptides derived from plants may mitigate excessive inflammatory responses by modulating inflammatory signaling and reducing the release of inflammatory factors [9,73]. Plant proteins are abundant in cereals, nuts, fruits, and other natural foods. Inflammation can be triggered by stimuli such as LPS, dextran sulfate sodium (DSS), and other toxins [108]. The MAPK and NF-κB pathways mediate inflammation. Inflammatory cytokines such as IL-1β, IL-6, and TNF-α contribute to intestinal and systemic inflammatory responses in conditions such as IBD and obesity, and may also be involved in dysbiosis-related gut immune activation observed in some cases of SIBO [89]. Although inflammatory signaling is relevant to several gut disorders, these pathways should not be taken as direct evidence that peptide interventions improve SIBO unless SIBO is measured using accepted clinical endpoints. Bioactive peptides may help control inflammation by acting on MAPK pathways. The MAPK family comprises a family of intracellular serine/threonine protein kinases involved in the phosphorylation of target proteins at serine and threonine residues [109]. The three branches of the MAPK family, extracellular signal-regulated protein kinases (ERK), c-Jun *N*-terminal kinase (JNK), and p38 MAPK (p38), play vital roles in stress responses, such as inflammation and apoptosis. These protein kinases are phosphorylated upon stimulation by inflammatory stimuli in cells.

Bioactive peptides from plants may reduce inflammatory processes by inhibiting ERK and p38 phosphorylation. For example, millet-derivedpeptides in the form of millet have been shown to inhibit the phosphorylation of ERK, JNK, and p38 [78]. In another study, wheat peptides suppressed MAPK signaling by decreasing the phosphorylation of ERK and p38 [101]. The NF-κB pathway is a key mediator of inflammation and regulates nitric oxide synthase (iNOS), IL-1β, IL-6, and TNF-α. Inflammatory factors serve as crucial indicators for many diseases, and their levels can be used to estimate disease incidence and characterize disease activity in the body. Monitoring and modulating inflammatory factors are essential for treating inflammation and chronic diseases. The NF-κB pathway has been extensively studied to identify target sites for effective regulation. One primary target site for plant-based bioactive peptides is the transcription factor p65. Research has shown that plant-derived peptides from cereals, nuts, and other plant sources can inhibit the release of inflammatory cytokines by blocking the translocation of p65, which is found in cereals, nuts, and other plants [90,110]. Moreover, clinical nutritional interventions in childhood undernutrition are summarized in Table 2. All entries fall into category (v) as defined in Section 2.4: microbiota-directed complementary food (MDCF-2), bioactive glycans, oat-containing ready-to-use therapeutic food, and rice bran-fortified therapeutic food are complex food matrices in which no peptide fraction was isolated or linked to the reported outcome. They describe the clinical context in which a peptide-based approach would have to operate, and no inference is drawn from them about the efficacy of plant-derived peptides. In particular, the growth and microbiota benefits reported for MDCF-2 are not attributed, in whole or in part, to peptides. The gut-targeted nutritional interventions and proposed mechanistic approaches that may modulate SIBO-relevant mechanisms in malnourished children are summarized in Table 2.

### 5.4. Improved Nutrient Absorption

Undernourished children with SIBO often experience nutrient malabsorption. Plant-derived protein hydrolysates and peptides may influence nutrient absorption indirectly by improving barrier integrity, modulating microbiota-derived SCFA production, and, for transportable di- and tripeptide fragments, using PEPT1-mediated transport, thereby supporting energy balance and growth. SIBO is prevalent in undernourished children and frequently results in compromised nutrient absorption due to bacterial competition and damage to the intestinal lining. Excess bacteria in the small intestine can have additional adverse effects beyond these symptoms, including malabsorption of specific nutrients, such as vitamin B12, steatorrhea, and deficiencies in fat-soluble vitamins [122]. Plant protein peptides may influence mechanisms relevant to SIBO by altering microbiota composition, although no included study measured bacterial density in the small intestine or applied a diagnostic test for SIBO. Some peptides also improve gut barrier function, reducing the inflammation and permeability that accompany bacterial overgrowth in experimental models. By enhancing the integrity of the intestinal lining, peptides may improve digestion and nutrient absorption of proteins, vitamins, and minerals [9]. They also exhibit prebiotic-like actions that favor short-chain fatty acid synthesis, thereby potentially increasing nutrient and energy availability. Enhanced gut function may support weight gain, growth, and recovery from undernutrition in children. Therefore, plant protein peptides could be explored as functional ingredients for targeting gut dysfunction in undernutrition and enhancing nutritional outcomes.

Available evidence from transporter physiology further indicates that transportable di- and tripeptide fragments may enhance nutrient absorption through PEPT1-mediated transport of di- and tripeptides, a process central to efficient protein utilization. Di- and tripeptides, along with peptide-like compounds, are absorbed through proton-dependent oligopeptide transporters, including PEPT1, PEPT2, PHT1, and PHT2, in intestinal and renal epithelial cells [20]. Mineral chelation is discussed in Section 4.7. The available evidence concerns casein-derived phosphopeptides rather than plant-derived preparations [123,124,125] and is not attributed to the intervention class reviewed here. Additionally, bioactive peptides may influence gut microbiota, immunity, and gut–brain axis function [87]. Microbiota-derived neuroactive compounds such as GABA, serotonin, dopamine, and ACh may mediate bidirectional communication between the gastrointestinal system and the brain in response to dietary phytochemicals, while the vagus nerve provides a physical communication route between these systems [126]. Plant peptides have also been associated with cognitive improvement, memory enhancement, and anxiolytic and antidepressant activities, mainly outside the context of childhood undernutrition. Nevertheless, these mechanisms remain biologically plausible rather than clinically established in undernourished children.

A potential mechanism by which transportable peptide fragments could promote nutrient uptake is di- and tripeptide transport via PEPT1, allowing the uptake of amino acids in a less energy-demanding form [127]. Moreover, these peptides may complement the activity of brush-border peptidases and disaccharidases, making carbohydrates and proteins easier to break down at the mucosal surface. Enhanced enzyme activity may increase nutrient availability for absorption [128]. In addition, plant peptides may contribute to shaping the structure of the gut microbiota and promote the generation of SCFAs, such as acetate, propionate, and butyrate. SCFAs are important energy sources for colonocytes, aid in mucosal healing, and help preserve intestinal barrier integrity. They may alleviate contributors to malabsorption associated with SIBO by reducing gut permeability and inflammation, although direct SIBO endpoints have not been measured for the plant-derived peptide interventions reviewed here. Together, improved peptide transport, enzyme activity, and SCFA generation could support energy balance, growth, and recovery from undernutrition [129] (Table 3).

## 6. Limitations and Future Directions

Plant-derived protein hydrolysates and bioactive peptides may support intestinal barrier integrity, microbiota composition, inflammatory signaling, and nutrient absorption in childhood undernutrition, acting through regulation of TJ proteins, shifts in microbial community composition, suppression of inflammatory pathways, and proposed peptide-transport and mineral-chelating mechanisms. These conclusions should be interpreted cautiously, however, because the evidence derives largely from in vitro and animal studies rather than from pediatric clinical trials.

Translation into large-scale interventions remains limited by poor oral bioavailability, gastrointestinal degradation, limited permeability across biological barriers, and low peptide concentrations in target tissues [25,26,29]. Peptide-based enteral formulas appear clinically useful in undernourished children with compromised gastrointestinal function, having been associated with improved tolerance and absorption, better nitrogen balance, reduced diarrhea and bacterial translocation, and restoration of gut integrity [130]. *Legume*-supplemented feeds have also been studied for severe acute malnutrition [131], but controlled trials examining plant-derived peptides in undernutrition-associated SIBO remain scarce. Future research should prioritize pediatric clinical trials evaluating plant protein-derived peptides within ready-to-use therapeutic foods, with endpoints covering gut integrity, microbiota balance, nutrient absorption, and growth recovery. Advanced delivery approaches, including nanoencapsulation, lipid–peptide conjugates, and polymeric systems, may protect peptides from enzymatic degradation and improve targeted absorption [27,108], while omics and bioinformatics tools could clarify peptide–microbiota–host interactions. However, most peptide candidates have been evaluated for broad antioxidant or barrier-protective activity rather than for engagement with specific anti-inflammatory enzymes or signaling axes. Therefore, future studies should include defined host-signaling endpoints. For instance, 12R-HETE has recently been identified as an endogenous Nur77 ligand regulating NKp46^+^ ILC3 development in the intestine [132].

Two further limitations concern the evidence base itself. First, in most studies, the intervention administered was a whole hydrolysate rather than a defined sequence, so structure–activity relationships cannot be established, reproducibility is compromised because a hydrolysate is defined by its preparation rather than its composition, and regulatory translation is hindered because a therapeutic preparation requires a specified active component. Second, no included study administered a plant-derived peptide in an undernutrition model; the intestinal findings come predominantly from chemically induced colitis, which reproduces barrier disruption and mucosal inflammation but not nutrient deficiency or growth faltering. The relevance of these mechanisms to undernourished children is therefore an assumption of this review rather than a finding of the primary literature. Priority should accordingly be given to studies that isolate, sequence, and independently test the active fraction, and that evaluate it in models of undernutrition.

## 7. Conclusions

This review indicates that plant-derived protein hydrolysates and bioactive peptides may support gut health in undernutrition through barrier protection, inflammatory modulation, oxidative-stress regulation, microbiota-related effects, and nutrient-transport mechanisms. However, the evidence is mainly mechanistic and preclinical, and current studies do not establish that peptide interventions reduce clinically diagnosed SIBO. Future trials should include clearly defined SIBO endpoints, such as standardized breath testing or small-bowel aspirate culture where feasible, alongside gut permeability, inflammatory, oxidative-stress, microbiome, growth, and safety outcomes. Moreover, innovative therapies aimed at restoring gut microbial equilibrium in undernourished individuals with SIBO, such as microbiota transplantation, targeted combinations of probiotics and peptides, and precision nutrition strategies, are promising approaches that warrant careful clinical evaluation. To validate their therapeutic potential and support the development of peptide-enriched functional foods or nutraceutical interventions for improving gastrointestinal health in at-risk populations, further clinical trials, mechanistic studies, and assessments of peptide bioavailability are essential. At present, plant-derived peptides should be considered promising functional ingredients rather than clinically validated treatments for SIBO or undernutrition-related gut dysfunction. Additionally, future investigations should aim to enhance extraction and purification methods, standardize delivery systems, and conduct comprehensive clinical trials to evaluate the health benefits and safety of plant-derived peptides.

## Figures and Tables

**Table 1:** Plant protein sources, extraction methods, structural characteristics, evidence models, and reported bioactivities of plant-derived protein hydrolysates and peptides.

Plant Source	Extraction/Preparation	Peptide Type	Molecular Weight	Identified Sequences	Experimental Model	Analytical Method	Main Gut-Relevant Finding	Evidence Category (i–v)	References
Oat	Enzymatic hydrolysis of oat protein	Oat peptides (OPs)	<5 kDa	266 peptides identified; WGVGVRAERDA identified as the primary bioactive sequence	DSS-induced colitis in mice and Caco-2 cells	UPLC-MS/MS; PEAKS Studio 10.6; PeptideRanker; ELISA; RT-qPCR and Western Blotting (WB); 16S rRNA	Preserved the intestinal barrier, modulated the Keap1–Nrf2 axis, and altered gut bacterial composition; mitigated colitis symptoms	(iii)	[45]
Rice	Hydrolyzed with 0.2% trypsin	Rice protein peptides	<2.2 kDa	RVIEPR, LQEQEQGQVQS, and ERYQEQGYQ	DSS-induced colitis in mice	HPLC (MW distribution); LC-MS/MS; SEM; Western blot; RT-qPCR; ELISA; 16S rRNA sequencing; ZDOCK molecular docking	Alleviated DSS colitis via the Keap1–Nrf2 pathway with microbiota regulation	(ii)	[46]
Foxtail millet	Defatted flour treated with α-amylase, pepsin followed by pancreatin; lyophilized	Foxtail millet protein hydrolysate (FMPH)	1–3 kDa 60.3%; 3–5 kDa 21.5%; 5–10 kDa 17.7%	2620 peptides; abundant sequences included ANPYLQQPF, AYWQHQQLLP, and TLPFQQYWTPR	DSS-induced colitis and LPS-stimulated Caco-2 monolayers	Folin method; OPA assay; HPLC; amino acid analyzer; UPLC-MS/MS; PeptideRanker and BIOPEP; TEER; ELISA; RT-qPCR; Western blotting; immunofluorescence; HE staining; MPO assay	↓ Serum LPS ↓ NF-κ B p65 phosphorylation; ↑ ZO-1 and occludin; ↓ colonic IL-6, TNF-α, IL-17, and IFN-γ; ↓ CD4, IL-17, Th17 cells; ↓ NLRP3, ASC, cleaved caspase-1 and IL-1β; restored LPS-lowered TEER in Caco-2 cells	(iii)	[47]
Wheat	Commercially prepared wheat peptide	Wheat peptide (WP)	<500 Da 37.82%; 0.5–1 kDa 30.92%; 1–5 kDa 28.53%	313 peptides identified; abundant sequences included GIIQPQQPAQLEVIR, LQPFPQPQLPY, and QPPFSQQQQPPFSQQ	DSS-induced colitis and Caco-2 cells	Amino acid autoanalyzer; HPLC; LC-MS with PEAKS Studio and Byonic; HE, AB-PAS staining; IHC; ELISA; RT-qPCR; Western blotting; immunofluorescence; 16S rRNA sequencing; GC-MS; molecular docking	Restored goblet cells and mucus with increased MUC2; ↑ ZO-1, occludin, and claudin-1; ↓ colonic and serum IL-1β, IL-6, and TNF-α; ↓ MDA, ↑ SOD and T-AOC; ↓ Keap1 and ↑ Nrf2; ↑ *Desulfovibrio* and *Odoribacter* with ↓ *Bacteroides*; ↑ cecal acetic, propionic, and butyric acids	(iii)	[48]
Corn gluten meal	Sequential hydrolysis: Alcalase followed by Protamex; lyophilized	Corn protein hydrolysate with glutamine-rich peptides (APH)	Not reported; glutamine content 12.53%	NR	DSS-induced ulcerative colitis	Glutamate biosensor analyzer; HE histopathology; ELISA; 16S rRNA sequencing	↓ Serum diamine oxidase, ↓ intestinal permeability; ↓ colon shortening, DAI, and inflammatory cell infiltration; ↓ serum TNF-α, IL-6, and IL-1β; ↑ IL-10; ↑ colonic GSH-Px and SOD; ↑ *Lactobacillus* and *Pediococcus*; ↓ *Bacteroidota*	(iii)	[49]
Selenium-enriched soybean	Enzymatic hydrolysis of selenium-enriched 7S protein using Neutrase and Alcalase	Selenium-containing soybean peptides	<1 kDa; selenium content 40 µg/g	NR	DSS-induced ulcerative colitis	SA-50 for blood selenium; ELISA; BCA assay; HE staining; immunofluorescence; GC; 16S rRNA; Spearman correlation	↑ ZO-1 and occludin, ↑ colonic GSH-Px and T-SOD, and normalized colonic TNF-α, IL-6, and IL-10; ↑ cecal acetic and butyric acids; improved microbial α-diversity and the *Firmicutes*/*Bacteroidetes* ratio; ↑ *Lachnospiraceae* and *Lactobacillus*	(ii)	[50]
Soybean	Commercial soy hydrolysate; VPY subsequently synthesized	PEPT1-transportable tripeptide (VPY)	377.5 Da	VPY	Caco-2 monolayers, HT-29 cells, THP-1 macrophages; DSS colitis	RP-HPLC; LC-MS/MS; ELISA; WST-1 viability; H&E with blinded histologic scoring; MPO assay; real-time RT-PCR	↓ Reduced TNF-α-induced IL-8; no effect in PEPT1-negative HT-29 cells, ↑ PEPT1 mediation; ↓ weight, ↓ colonic TNF-α, IL-1β and IFN-γ mRNA	(i)	[51]
Wheat germ	Enzymatic digestion of wheat protein	Octapeptide	Octapeptide	APEPEPAF	DSS-induced colitis	Histopathology; ELISA; RT-qPCR; Western blotting; 16S rRNA sequencing	Alleviated weight loss, colon shortening and histopathological changes; ↓ inflammatory cytokine generation by inhibiting PKCζ (Thr410) phosphorylation and NF-κB transcriptional activity; ↑ Claudin-1, ZO-1, and occludin expression; ↓ *Bacteroides*; ↑ *Dubosiella,* and *Lachnospiraceae*	(i)	[52]
Walnut	Enzymatic digestion of walnut protein	Walnut protein peptides	<1 kDa (SHTLP 553.6 Da; HYNLN 659.7 Da; LGTYP 549.6 Da)	SHTLP, HYNLN, and LGTYP,	DSS-induced ulcerative colitis	LC-MS/MS; RT-qPCR; 16S rRNA sequencing with LEfSe; virtual screening and network pharmacology; molecular docking	Attenuated intestinal mucosal barrier dysfunction; ↓ inflammation by inhibiting activation of the TLR4-MAPK pathway; restored intestinal microbial homeostasis by reducing *Helicobacter* and *Bacteroides* and ↑ *Candidatus Saccharimonas*	(ii)	[53]

Abbreviations used: AB-PAS, Alcian Blue–Periodic Acid Schiff (staining); APH, corn protein hydrolysate with glutamine-rich peptides; ASC, apoptosis-associated speck-like protein containing a CARD; BCA, bicinchoninic acid (assay); CD4, cluster of differentiation 4; DAI, disease activity index; DSS, dextran sulfate sodium; ELISA, enzyme-linked immunosorbent assay; FMPH, foxtail millet protein hydrolysate; GC, gas chromatography; GC-MS, gas chromatography–mass spectrometry; GSH-Px, glutathione peroxidase; HE, hematoxylin and eosin (staining); HPLC, high-performance liquid chromatography; IFN-γ, interferon gamma; IHC, immunohistochemistry; IL-1β, interleukin-1 beta; IL-6, interleukin-6; IL-10, interleukin-10; IL-17, interleukin-17; Keap1, Kelch-like ECH-associated protein 1; LC-MS/MS, liquid chromatography–tandem mass spectrometry; LEfSe, linear discriminant analysis effect size; LPS, lipopolysaccharide; MAPK, mitogen-activated protein kinase; MDA, malondialdehyde; MOE, molecular operating environment (software); MPO, myeloperoxidase; MUC2, mucin 2; MW, molecular weight; NF-κB, nuclear factor kappa-light-chain-enhancer of activated B cells; NLRP3, NOD-, LRR-, and pyrin domain-containing protein 3; Nrf2, nuclear factor erythroid 2-related factor 2; OPA, o-phthalaldehyde (assay); OPs, oat peptides; PEPT1, peptide transporter 1; PKCζ, protein kinase C zeta; RP-HPLC, reversed-phase high-performance liquid chromatography; RT-qPCR, reverse transcription quantitative polymerase chain reaction; rRNA, ribosomal RNA; SEM, scanning electron microscopy; SOD, superoxide dismutase; T-AOC, total antioxidant capacity; TEER, transepithelial electrical resistance; Th17, T-helper 17 cells; Thr410, threonine residue at position 410; TLR4, Toll-like receptor 4; TNF-α, tumor necrosis factor-α; UPLC-MS/MS, ultra-performance liquid chromatography–tandem mass spectrometry; WB, Western blotting; WP, wheat peptide; WST-1, water-soluble tetrazolium salt-1; ZO-1, zonula occludens-1. All peptide sequences listed in this table are given in the standard single-letter amino acid code. Evidence categories (i)–(v) are defined in Section 2.4. Intervention names in the “Peptide type” column are those used in the primary studies; in the text, each intervention is named according to its evidence category.

**Table 2:** Non-peptide nutritional interventions in childhood undernutrition: contextual clinical studies.

Type of Intervention	Molecular/Cellular Target	Gut-Relevant Mechanistic Implication	Impact in Undernourished Children	Reference
Microbiota repair	Immature gut community structure; growth-discriminatory taxa	Shifted gut microbiota toward a more age-appropriate profile, suggesting potentially improved colonization resistance and gut ecosystem stability	Supported weight gain and improved growth-associated host biomarkers in children with MAM	[9]
Gut-health biomarker improvement	Plasma citrulline; stool/plasma environmental enteric dysfunction biomarkers	Higher citrulline suggests improved enterocyte mass/function and better intestinal health; relevant to reduced leakiness and dysbiosis burden	Suggested better gastrointestinal recovery in children with MAM	[111]
Post-severe acute malnutrition microbiome-directed recovery	*Prevotella copri* strains; fecal metagenome-assembled genomes; plasma proteome	Promoted microbiome reassembly after SAM treatment and enriched pathways linked to healthier gut-host metabolic interaction	Faster WLZ improvement and stronger recovery after SAM	[112]
Food-glycan–microbiome interaction	Bioactive glycans in MDCF-2; glycan utilization pathways	Identified food components that selectively feed beneficial microbes, supporting a more resilient microbial ecosystem	Helps explain why MDCF-2 improved growth more effectively than standard RUSF	[113]
Microbe-mediated host metabolic signaling	*Prevotella copri* and interacting microbiota members	Mechanistically linked targeted microbes with metabolism of MDCF-2 glycans and downstream host metabolic benefits, relevant to correcting dysbiosis	Provides a biological explanation for improved recovery in malnourished children receiving MDCF-2	[114]
Early proof-of-concept microbiota targeting	Gut microbiota immaturity; plasma growth mediators	Reported microbiota repair with concurrent improvement in host biomarkers, consistent with better gut functional recovery	Proof-of-concept study indicated weight gain and a healthier biological profile in MAM	[115]
Barrier integrity testing	Intestinal permeability measured by lactulose permeability testing	Oat-containing RUTF did not reduce permeability versus standard RUTF, showing that this formulation’s benefit was not mediated through this barrier marker	Clinical recovery improved in a related efficacy trial, but permeability itself did not improve	[116]
Clinical recovery with cereal-enriched therapeutic food	Whole-gut response to oat-containing RUTF	Suggests that nutritional rehabilitation can improve outcomes even when one gut-barrier marker is unchanged	Oat-RUTF improved SAM recovery compared with standard RUTF	[116]
Microbiota modulation	Stool microbiota composition; metabolome signatures	Rice bran shifted microbiota and stool metabolites in ways consistent with a more favorable gut environment	Improved LAZ in infants at risk of malnutrition	[117]
Mucosal immunity and enteric dysfunction	Fecal sIgA, myeloperoxidase, α1-antitrypsin, neopterin, EED score	Rice bran lowered the EED score and maintained sIgA, supporting improved mucosal defense and less enteric dysfunction	Relevant for children vulnerable to undernutrition and diarrhea	[118]
Prebiotic enrichment of therapeutic food	Rice bran as a fermentable substrate for microbiota	The trial was built on the premise that stabilized rice bran may support gut microbiota and gut recovery during treatment	Rice bran-fortified RUTF supported better early weight and MUAC velocity	[119]
Microbiota-directed complementary feeding feasibility	Gut microbiota-targeted food design	Did not report full mechanistic biomarker outcomes, but established feasibility and safety of microbiota-directed feeding in MAM	Supports the scalability of gut-targeted nutrition trials in malnourished children	[120]
Microbiome-directed food acceptability	Gut microbiota-repairing formulation	Did not measure mechanistic biomarkers directly, but showed that a microbiome-directed food could be delivered and accepted in acute malnutrition programs	Supports the practical use of gut-targeted foods in children with SAM and MAM	[121]

Abbreviations used: AAT, α1-antitrypsin; EED, environmental enteric dysfunction; LAZ, length-for-age z-score; MAM, moderate acute malnutrition; MDCF-2, microbiota-directed complementary food-2; MPO, myeloperoxidase; MUAC, mid-upper arm circumference; RUSF, ready-to-use supplementary food; RUTF, ready-to-use therapeutic food; SAM, severe acute malnutrition; sIgA, secretory immunoglobulin A; WLZ, weight-for-length z-score.

**Table 3:** Evidence level and evidence-strengthsummary of proposed mechanisms linking plant-derived protein hydrolysates and peptides and gut health in childhood undernutrition.

Mechanism	Evidence Type	Model/Population	Intervention/Comparator	Baseline Adjustment	Main Outcome	Major Limitation	Evidence Strength	Reference
Intestinal barrier restoration via tight-junction proteins (occludin, claudins, ZO-1)	Preclinical (cell culture, mechanistic in vivo models)	Intestinal epithelial cell models; animal gut barrier models	Plant-derived protein hydrolysates and peptides vs. untreated or disease control	Not consistently reported across studies	Improved barrier integrity, reduced permeability, and increased TJ protein expression	Heterogeneity in peptide sources; limited human trials	Moderate	[45,46,47,48]
Modulation of gut microbiota composition	Preclinical and limited clinical observations	Gut microbiota models; dysbiosis contexts in undernutrition	Plant protein hydrolysates and peptides vs. baseline dysbiosis/no intervention	Rarely adjusted for baseline differences; mainly experimental studies	Increased commensal taxa (*Bifidobacteria*, *Lactobacillus*), reduced pathobiont abundance	Compositional shifts only; no direct antibacterial testing	Moderate	[54,67,87]
Anti-inflammatory pathway modulation (NF-κB, IL-6, TNF-α)	Preclinical (cellular and animal models)	Intestinal inflammation models	Plant protein hydrolysates and peptides vs. control diet or untreated inflammation models	Partially controlled in animal studies	Reduced pro-inflammatory cytokines and inhibited NF-κB signaling	Limited translation to human inflammatory gut disease	Low-Moderate	[20,21]
Reduction in intestinal oxidative stress markers	In vivo animal studies	DSS-induced colitis in mice	Hydrolysate- or peptide-treated vs. DSS control	Not adjusted; group comparisons against a concurrent DSS control without stratification for baseline redox status	Reduced colonic malondialdehyde; increased superoxide dismutase, catalase, glutathione peroxidase and total antioxidant capacity	Colitis models only; no undernutrition model; doses not directly comparable across studies	Moderate	[45,46,48,49,50]
Keap1–Nrf2–ARE pathway activation	In vitro and in vivo	Caco-2 monolayers and colitic mouse colon	Hydrolysate- or peptide-treated vs. untreated or DSS control	Not adjusted; Caco-2 comparisons against untreated controls, with no baseline pathway measurement	Reduced Keap1 and increased nuclear Nrf2; induction of HO-1, NQO1, and Gclc	Pathway measured predominantly in Caco-2 cells; three studies only; no undernutrition model	Low–Moderate	[45,46,48]
Enhanced nutrient absorption via the PEPT1 transport system	Preclinical (cell transport models)	Intestinal epithelial transport models	Peptide exposure vs. control transport conditions	Not reported	Increased di-/tripeptide uptake via PEPT1 transporter	No included study measured PEPT1 expression or transport of a plant-derived peptide	Low	[20,21]
Direct antibacterial activity of food-derived plant peptides	No direct evidence identified for the intervention class reviewed	Not represented in any included study	No food-derived plant hydrolysate or peptide was tested against a bacterial growth endpoint	Not applicable	Not measured. Minimum inhibitory concentration data exist only for gene-encoded plant antimicrobial peptides (defensins, thionins, lipid-transfer proteins, cyclotides), which are a different intervention class	No included study reported minimum inhibitory concentration, time-kill, or zone-of-inhibition data; reported pathogen suppression rests on compositional shifts	Insufficient	[69,71,72]
Gut–brain axis modulation	Single preclinical study	DSS-induced colitis in mice; no undernutrition model	Wheat protein hydrolysate at 200 and 500 mg/kg vs. DSS control	Not adjusted; concurrent DSS control	Reversal of open field test deficits alongside restored tight-junction proteins and reduced colonic and serum cytokines	One study, one behavioral endpoint; no neurotransmitter, vagal, or cognitive measurement in undernutrition	Low	[48]

Abbreviations used: ARE, antioxidant response element; DSS, dextran sulfate sodium; Gclc, glutamate-cysteine ligase catalytic subunit; HO-1, heme oxygenase-1; IL-6, interleukin-6; Keap1, Kelch-like ECH-associated protein 1; NF-κB, nuclear factor kappa-light-chain-enhancer of activated B cells; NQO1, NAD(P)H quinone dehydrogenase 1; Nrf2, nuclear factor erythroid 2-related factor 2; PEPT1, peptide transporter 1; SIBO, small intestinal bacterial overgrowth; TJ, tight junction; TNF-α, tumor necrosis factor alpha; ZO-1, zonula occludens-1.

## Data Availability

No new data were created or analyzed in this study. Data sharing is not applicable to this article.

## Abbreviations

The following abbreviations are used in this manuscript:

ACE	Angiotensin-converting enzyme
ACh	Acetylcholine
AMPs	Antimicrobial peptides
aPKC	Atypical protein kinase C
ARE	Antioxidant response element
*C. elegans*	*Caenorhabditis elegans*
CNS	Central nervous system
COX-2	Cyclooxygenase-2
DPP-IV	Dipeptidyl peptidase IV
ENS	Enteric nervous system
ERK	Extracellular signal-regulated kinase
GABA	Gamma-aminobutyric acid
GALT	Gut-associated lymphoid tissue
GBA	Gut–brain axis
GIT	Gastrointestinal tract
HO-1	Heme oxygenase-1
HPA	Hypothalamic–pituitary–adrenal axis
IL-1β	Interleukin-1 beta
IL-6	Interleukin-6
IL-8	Interleukin-8
IL-10	Interleukin-10
JAMs	Junctional adhesion molecules
JNK	c-Jun *N*-terminal kinase
Keap1	Kelch-like ECH-associated protein 1
LAT1/SLC7A5	Large neutral amino acid transporter 1/solute carrier family 7 member 5
LAZ	Length-for-age Z-score
LC-ESI-MS/MS	Liquid chromatography–electrospray ionization tandem mass spectrometry
LC-MS/MS	Liquid chromatography–tandem mass spectrometry
MAGs	Metagenome-assembled genomes
MAM	Moderate acute malnutrition
MAPKs	Mitogen-activated protein kinases
Mas1	MAS receptor
MDA	Malondialdehyde
MDCF-2	Microbiota-directed complementary food prototype 2
MUAC	Mid-upper arm circumference
NF-κB	Nuclear factor kappa B
NO	Nitric oxide
NQO1	NAD(P)H quinone oxidoreductase 1
NR	Not reported
Nrf2	Nuclear factor erythroid 2-related factor 2
PC12	Pheochromocytoma 12 cell line
PEPT1/POT	Peptide transporter 1/proton-dependent oligopeptide transporter
PKA	Protein kinase A
ROS	Reactive oxygen species
RUSF	Ready-to-use supplementary food
RUTF	Ready-to-use therapeutic food
SCFAs	Short-chain fatty acids
SDGs	Sustainable development goals
SDS-PAGE	Sodium dodecyl sulfate-polyacrylamide gel electrophoresis
SIBO	Small intestinal bacterial overgrowth
sIgA	Secretory immunoglobulin A
SOD	Superoxide dismutase
TJ(s)	Tight junction(s)
TNF-α	Tumor necrosis factor-alpha
UPLC-QTRAP LC-MS	Ultra-performance liquid chromatography coupled with quadrupole linear ion trap mass spectrometry
WEKPPVSH	Walnut protein hydrolysate-derived peptide
WLZ	Weight-for-length Z-score
ZOs	Zonula occludens proteins
